# System Review about Function Role of ESCC Driver Gene KDM6A by Network Biology Approach

**DOI:** 10.1155/2016/1970904

**Published:** 2016-05-17

**Authors:** Jihua Ran, Hui Li, Huiwu Li

**Affiliations:** ^1^Department of Biochemistry and Molecular Biology, School of Basic Medicine, Xinjiang Medical University, 393 Xinyi Road, Urumqi, Xinjiang 830054, China; ^2^Clinical Laboratory Diagnosis Center of PLA, Urumqi General Hospital, 359 Youhao Road, Urumqi, Xinjiang 830054, China; ^3^Central Laboratory of Xinjiang Medical University, 393 Xinyi Road, Urumqi, Xinjiang 830054, China; ^4^Cancer Institute, The Affiliated Cancer Hospital of Xinjiang Medical University, 789 East Suzhou Road, Urumqi, Xinjiang Uyghur Autonomous Region 830011, China

## Abstract

*Background.* KDM6A (Lysine (K)-Specific Demethylase 6A) is the driver gene related to esophageal squamous cell carcinoma (ESCC). In order to provide more biological insights into KDM6A, in this paper, we treat PPI (protein-protein interaction) network derived from KDM6A as a conceptual framework and follow it to review its biological function.* Method.* We constructed a PPI network with Cytoscape software and performed clustering of network with Clust&See. Then, we evaluate the pathways, which are statistically involved in the network derived from KDM6A. Lastly, gene ontology analysis of clusters of genes in the network was conducted.* Result.* The network includes three clusters that consist of 74 nodes connected via 453 edges. Fifty-five pathways are statistically involved in the network and most of them are functionally related to the processes of cell cycle, gene expression, and carcinogenesis. The biology themes of clusters 1, 2, and 3 are chromatin modification, regulation of gene expression by transcription factor complex, and control of cell cycle, respectively.* Conclusion.* The PPI network presents a panoramic view which can facilitate for us to understand the function role of KDM6A. It is a helpful way by network approach to perform system review on a certain gene.

## 1. Introduction

Esophageal cancer is the eighth most common malignancy and the sixth cause of cancer deaths worldwide [1, 2]. Esophageal squamous cell carcinoma (ESCC) and adenocancer are the two histologic types that make up for greater than 90 percent of the diagnoses of esophageal cancers [3]. In East Asia, the majority of esophageal cancers are ESCC [2]. As the most of cancers, the pathomechanism of ESCC remains elusive. There is an agreement that cancers arise owing to mutations in a subset of genes that confer growth advantage. Not all mutations can contribute to the development of the cancers. The genes which harbor mutation conferring benefit to survival of tumor cell are called driver genes. The counterpart of driver gene is passenger gene, whose mutation does not contribute to oncogenesis [4]. Defining a driver gene in physiologic terms is easy but identifying which mutations are drivers and which are passengers is more difficult, so numerous statistical methods to identify driver genes have been described. Vogelstein and colleagues identified about 140 driver genes from Catalogue of Somatic Mutations in Cancer database by their unique ruler [5]. Of them, KDM6A is the driver gene related to ESCC.

In eukaryotic cells, DNA is packaged into chromatin whose functional unit is nucleosome. A nucleosome is an octameric structure composed of two histones each of H2A, H2B, H3, and H4 encircled by 147 bp of DNA [6]. Modifications of histones such as methylation, acetylation, phosphorylation, ubiquitination, and sumoylation regulate the structure of chromatin and determine the active or repressive chromatin states. Of them, histone methylation can function in gene activation or repression, depending on which residues are targeted. Methylation of histone H3 on Lysine 4 (H3K4me) is an active chromatin modification, while methylation of histone H3 on Lysine 27 (H3K27me) is associated with repression of gene activity [7]. The PRC2 (polycomb repressive complex 2) mediates transcriptional repression by methylation of histone on H3K27 [8–11]. KDM6A counteracts PRC2 and activates chromatin transcriptionally by demethylation of H3K27 [12]. This is the main biological function of KDM6A. However, KDM6A is vital in a wide array of functions including cell cycle regulation, cell differentiation, and stem cell specification [13–15]. The mutation of KDM6A is involved in various types of cancer across both solid and liquid tumors [16–18].

As we all know, proteins do not function alone while they orchestrate all biological processes by interacting with other proteins. Analysis of PPI network consisting of KDM6A and its interactors facilitates providing more biological insights into KDM6A for us. In this paper, we treat PPI network derived from KDM6A as a conceptual framework and follow it to review its biological function.

## 2. Method

### 2.1. Construction of PPI Network Derived from KDM6A

We obtained PPIs from STRING database, a precomputed database for the exploration of protein-protein interactions. The prediction methods in STRING include neighborhood gene fusion, cooccurrence, coexpression, experiments, databases, and text mining. A confidence score can be assigned to each prediction method and every interaction has a final aggregate score. The newest version of STRING 9.1 covers approximately 2.5 million proteins from 630 different organisms [19]. In this study, the interactions restricted to* Homo sapiens* were downloaded. In order to avoid false link in greatest extent, we set the score of inclusion criteria larger than 0.9. We constructed a network that consists of not only the direct PPI neighbors of KDM6A but also their secondary neighbors. The network was constructed using Cytoscape, a popular and highly versatile software platform for the analysis, operation, and visualization of large networks [20].

### 2.2. Construction of Cellular Pathways Database

All of the pathways with their *m* gene members were downloaded from an integrated pathway database, Molecular Signatures Database (MSigDB) [21], which is a large collection of annotated functional gene sets. There are 880 canonical pathways with 6804 proteins members in the database, including the metabolic and signaling pathways collected from BioCarta (http://www.biocarta.com/), KEGG [22], and Reactome [23].

### 2.3. Identification of Pathways Involved in PPI Network Derived from KDM6A

To examine the evidence of association of a given pathway with PPI network derived from KDM6A, Fisher's exact test based on the cumulative hypergeometric distribution was employed. The *p* value was calculated to evaluate statistical significance of a given pathway by the formula as follows:

(1) $$\begin{array}{c} p=1-\overset{k-1}{\underset{i=0}{\sum }}\frac{\left(\begin{array}{c} m\\ i \end{array}\right)\left(\begin{array}{c} N-m\\ n-i \end{array}\right)}{\left(\begin{array}{c} N\\ n \end{array}\right)}. \end{array}$$

In this formula, *N* represents the total number of proteins in the background population, *n* represents the number of proteins in the PPI network derived from KDM6A, and *m* denotes the number of proteins within the given pathways. The number of proteins that overlapped with both proteins in the PPI network and this pathway is denoted as *k*. In this study, a pathway is considered statistically associating with the PPI network derived from KDM6A under the condition of its *p* value being less than 0.05.

### 2.4. Ontology Analysis of Proteins in PPI Network Derived from KDM6A

Usually, gene ontology analysis is mainly conducted in individual gene. However, in this study, we perform ontology enrichment analysis based on a group of genes. As a web tool, NOA not only is able to perform analysis based on a group of genes but also takes interactions between genes into consideration [24]. Every group of genes corresponds to the proteins in the clusters of PPI network derived from KDM6A. There are many clustering methods that have been used to highlight groups of densely connected nodes [25]. Clusters in networks can offer mechanistic hypotheses of disease because they are highly interconnected molecular complexes or signaling pathways [26]. In this study, clusters were found with Cytoscape plugin called Clust&See dedicated to the identification, visualization, and analysis of clusters extracted from PPI network [27].

## 3. Result and Discussion

### 3.1. General Description of the PPI Network Derived from KDM6A

**Figure 1:**
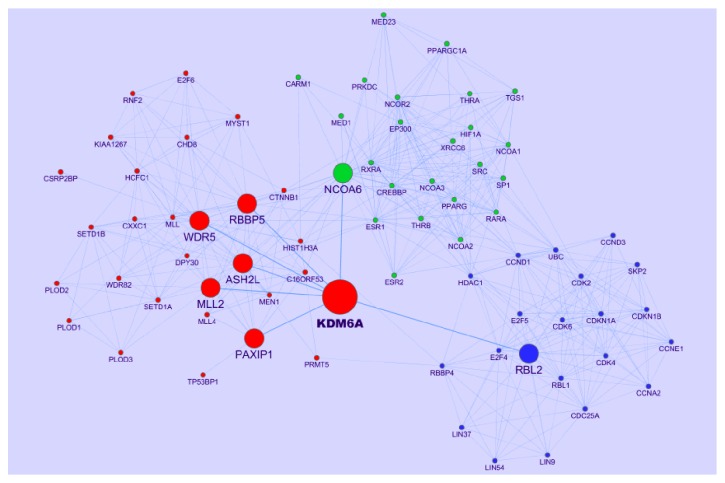
PPI network derived from KDM6A. KDM6A has seven direct neighbors tagged with middle node size. There are three clusters, whose nodes are distinguished with different colors (cluster 1: red; cluster 2: green; cluster 3: blue).

The network consists of 74 nodes connected via 453 edges. KDM6A has seven direct neighbors, which are RBBP5, WDR5, ASH2L, MLL2, PAXIP1, NCOA6, and RBL2, respectively (Figure 1). KDM6A contains a tetratricopeptide motif predicted to mediate protein-protein interactions [28] and is not only a member of a stable multiprotein complex that demethylates H3K27me3 but also a member of the MLL2 H3K4 methyltransferase complex which can facilitate gene expression [29, 30]. Its catalytic activity has been linked to regulation of homeobox (HOX) and RB transcriptional networks [31]. Different protein partners modulate the recruitment of KDM6A to specific chromatin regions to target specific genes [32, 33]. There are three clusters whose nodes are distinguished with different colors in the network (Figure 1). Every cluster has its biological theme detailed later and there are links among these clusters.

### 3.2. Pathways Involved in PPI Network Derived from KDM6A

**Figure 2:**
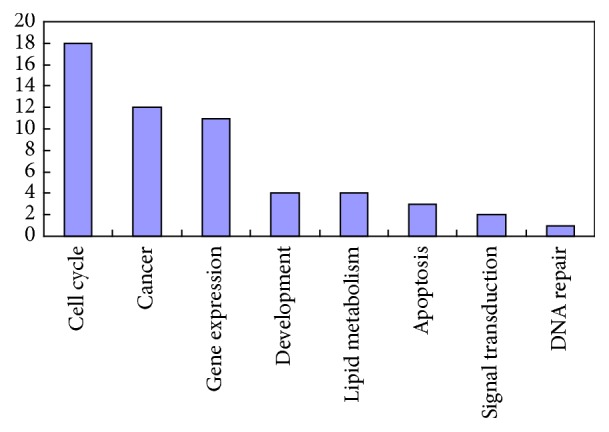
The frequency of fifty-five pathways. Fifty-five pathways can be catalogue to eight classes including cell cycle, gene expression, lipid metabolism, cancer, apoptosis, signal transduction, development, and DNA repair.

Of eight hundred and eighty (880) pathways, fifty-five (55) pathways are statistically involved in PPI network derived from KDM6A (*p* < 0.05, see Table 1 in Supplementary Material available online at http://dx.doi.org/10.1155/2016/1970904). These fifty-five pathways can be catalogue to eight classes including cell cycle, gene expression, lipid metabolism, cancer, apoptosis, signal transduction, development, and DNA repair. Most of pathways are related to cell cycle, gene expression, and cancer (Figure 2).

There are two points in current mainstream discipline about KDM6A: (i) as a general factor, KDM6A activates gene transcription during development. HOX genes encode transcription factors that regulate embryogenesis and guide tissue differentiation [34]. KDM6A removes H3K27me3 from HOX genes to restore their activity and control HOX gene expression [35]. (ii) KDM6A acts with methyltransferase complex to facilitate gene expression for regulating transcriptional networks of RB [36].

The evidence indicates that KDM6A evolved in pathways such as inflammation, apoptosis, cell cycle, and DNA repair related. KDM6A is implicated in IL-4 mediated transcriptional activation of the arachidonate 15-lipoxygenase-1 (ALOX15) gene. ALOX15 oxygenates polyunsaturated fatty acids and biomembranes, which generate multiple lipid signaling mediators involved in inflammation [37]. KDM6A is required for hormone-mediated transcriptional regulation of apoptosis and autophagy genes in* Drosophila* salivary glands [38]. DNA methylation affects the expression of genes involved in cell cycle checkpoint, apoptosis, and DNA repair [39].

### 3.3. Ontology Analysis of the Protein Interactions in the Three Clusters

We manually choose top three terms ranked by *p* value generated by analysis tool NOA (Table 1). The theme of cluster 1 is chromatin modification by methyl transfer of histone. It is the main function of KDM6A as previously mentioned. As part of an H3K4-methyltransferase complex containing MLL2, PTIP, ASC2, ASH2, RBQ3, and WDR5, KDM6A can promote H3K4 methylation [30]. It indicates that KDM6A and methyltransferase complex concert with active chromatin modification by demethylation of histone on H3K27 and methylation on K3K4. Clinically, KDM6A or MLL4 was associated with poor prognosis in patients with breast cancer. KDM6A interacts with a c-terminal region of MLL4 and coordinates regulation of cotarget gene expression [40].

The proteins in cluster 2 mainly compose the transcription factor complex. The direct neighbor of KDM6A, NCOA6 (nuclear receptor coactivator 6, NCOA6), binds nuclear receptors and stimulates the transcriptional activities in a hormone-dependent fashion. Mass spectrometry analysis demonstrated that PTIP associated with ASH2L, RBBP5, WDR5, NCOA6, and KDM6A [29]. It suggests that there exist interactions among NCOA6, KDM6A, and other methyltransferases.

The nodes in cluster 3 are RBL2 (retinoblastoma-like 2, RBL2) and its interactors which control cell cycle. RBL2 is a key regulator of entry into cell division, directly involved in heterochromatin formation by maintaining overall chromatin structure through stabilizing histone methylation. Even though there is no direct experimental evidence that KDM6A interacts with RBL2, a study found that ectopic expression of KDM6A enhanced the expression of retinoblastoma tumor suppressor gene RB and its related gene RBL2 [36].

## 4. Conclusion and Prospect

PPI network derived from KDM6A presents a panoramic view which can facilitate the understanding of the function role of KDM6A for us. The results from both sides of identification of pathway and ontology analysis of network confirm and complement one another. It is a helpful way by network approach to perform system review on a certain gene. Considering that these could be nonspecific interactions, the network can only be regarded as a guide map. Some links between the proteins in the network need to be validated by experiment to explore the biology role of KDM6A in the progression of carcinogenesis. For example, we need to investigate the expression pattern of these genes at mRNA or protein level and the possible regulatory relationships between these genes in clinical ESCC specimen or esophageal cancer cell line. Similar studies in other cancers were completed and reported [40, 41].

## Supplementary Material

List of pathways involved in PPI network derived from KDM6A.

## Figures and Tables

**Table 1:** The functional characterization of protein interaction networks in three clusters.

Cluster		GO:term	Term name	*p* value
Cluster 1	Biological processes	GO:0016568	Chromatin modification	7.9*E* − 21
		GO:0006325	Chromatin organization	1.3*E* − 20
		GO:0051568	Histone H3-K4 methylation	9.2*E* − 20
	Cellular components	GO:0034708	Methyltransferase complex	5.6*E* − 38
		GO:0035097	Histone methyltransferase complex	5.6*E* − 38
		GO:0044451	Nucleoplasm part	3.4*E* − 20
	Molecular functions	GO:0042054	Histone methyltransferase activity	2.5*E* − 20
		GO:0008170	N-Methyltransferase activity	5.6*E* − 19
		GO:0008276	Protein methyltransferase activity	5.6*E* − 19

Cluster 2	Biological processes	GO:0045944	Positive regulation of transcription from RNA polymerase II promoter	4.7*E* − 16
		GO:0006357	Regulation of transcription from RNA polymerase II promoter	4.6*E* − 15
		GO:0010628	Positive regulation of gene expression	9.5*E* − 15
	Cellular components	GO:0005634	Nucleus	3.4*E* − 9
		GO:0044451	Nucleoplasm part	6.2*E* − 9
		GO:0005667	Transcription factor complex	4.3*E* − 7
	Molecular functions	GO:0008134	Transcription factor binding	6.2*E* − 23
		GO:0016563	Transcription activator activity	1.6*E* − 18
		GO:0030528	Transcription regulator activity	9.6*E* − 16

Cluster 3	Biological processes	GO:0000082	G1/S transition of mitotic cell cycle	1.8*E* − 11
		GO:0007049	Cell cycle	9.7*E* − 10
		GO:0022402	Cell cycle process	5.1*E* − 9
	Cellular components	GO:0000307	Cyclin-dependent protein kinase holoenzyme complex	8.9*E* − 14
		GO:0005654	Nucleoplasm	4.3*E* − 10
		GO:0044428	Nuclear part	6.7*E* − 9
	Molecular functions	GO:0004693	Cyclin-dependent protein kinase activity	3.7*E* − 6
		GO:0016538	Cyclin-dependent protein kinase regulator activity	9.5*E* − 5
		GO:0030332	Cyclin binding	3.0*E* − 3
