# Supplementary material for: System Review about Function Role of ESCC Driver Gene KDM6A by Network Biology Approach
Source: ScientificWorldJournal. 2016 May 17;2016:1970904. doi: 10.1155/2016/1970904 (PMC4886093; doi:10.1155/2016/1970904)
Supplement: Supplementary file 1 — List of pathways involved in PPI network derived from KDM6A. [file 1970904.f1.doc]

Table 1 Pathways statistically involved in PPI network derived from KDM6A

| **SN** | **Pathway name** | **Number of overlapped proteins** | **P-value** | **Class ID*** |
| --- | --- | --- | --- | --- |
| **1** | KEGG_CELL_CYCLE | 19 | 3.699E-16 | 1 |
| **2** | BIOCARTA_VDR_PATHWAY | 9 | 3.106E-15 | 2 |
| **3** | BIOCARTA_CELLCYCLE_PATHWAY | 10 | 1.088E-14 | 1 |
| **4** | BIOCARTA_G1_PATHWAY | 10 | 1.344E-13 | 1 |
| **5** | SA_REG_CASCADE_OF_CYCLIN_EXPR | 7 | 2.145E-11 | 1 |
| **6** | BIOCARTA_CARM_ER_PATHWAY | 9 | 7.062E-11 | 2 |
| **7** | REACTOME_G1_PHASE | 7 | 1.393E-10 | 1 |
| **8** | REACTOME_REGULATION_OF_LIPID_METABOLISM_BY_  PEROXISOME_PROLIFERATOR_ACTIVATED_RECEPTOR_ALPHA | 10 | 6.945E-10 | 3 |
| **9** | BIOCARTA_CARM1_PATHWAY | 6 | 2.134E-09 | 2 |
| **10** | BIOCARTA_RARRXR_PATHWAY | 6 | 6.117E-09 | 2 |
| **11** | BIOCARTA_RACCYCD_PATHWAY | 7 | 7.348E-09 | 1 |
| **12** | REACTOME_NUCLEAR_RECEPTOR_TRANSCRIPTION_PATHWAY | 8 | 4.829E-08 | 2 |
| **13** | BIOCARTA_EGFR_SMRTE_PATHWAY | 5 | 5.732E-08 | 2 |
| **14** | BIOCARTA_PPARA_PATHWAY | 8 | 1.609E-07 | 3 |
| **15** | KEGG_PATHWAYS_IN_CANCER | 16 | 2.960E-07 | 4 |
| **16** | SA_G1_AND_S_PHASES | 5 | 3.602E-07 | 1 |
| **17** | BIOCARTA_PITX2_PATHWAY | 5 | 3.602E-07 | 2 |
| **18** | BIOCARTA_P53_PATHWAY | 5 | 5.195E-07 | 5 |
| **19** | KEGG_SMALL_CELL_LUNG_CANCER | 8 | 2.922E-06 | 4 |
| **20** | KEGG_PROSTATE_CANCER | 8 | 4.533E-06 | 4 |
| **21** | KEGG_P53_SIGNALING_PATHWAY | 7 | 8.317E-06 | 5 |
| **22** | BIOCARTA_P27_PATHWAY | 4 | 8.448E-06 | 1 |
| **23** | REACTOME_S_PHASE | 8 | 1.352E-05 | 1 |
| **24** | REACTOME_CYCLIN_E_ASSOCIATED_EVENTS  _DURING_G1_S_TRANSITION_ | 6 | 3.422E-05 | 1 |
| **25** | BIOCARTA_ETS_PATHWAY | 4 | 3.470E-05 | 2 |
| **26** | KEGG_TGF_BETA_SIGNALING_PATHWAY | 7 | 3.577E-05 | 6 |
| **27** | REACTOME_CELL_CYCLE_MITOTIC | 12 | 9.533E-05 | 1 |
| **28** | KEGG_LYSINE_DEGRADATION | 5 | 1.020E-04 | 2 |
| **29** | BIOCARTA_G2_PATHWAY | 4 | 1.147E-04 | 1 |
| **30** | KEGG_CHRONIC_MYELOID_LEUKEMIA | 6 | 1.266E-04 | 4 |
| **31** | BIOCARTA_SKP2E2F_PATHWAY | 3 | 1.389E-04 | 1 |
| **32** | REACTOME_METABOLISM_OF_LIPIDS_AND_LIPOPROTEINS | 10 | 1.560E-04 | 3 |
| **33** | BIOCARTA_WNT_PATHWAY | 4 | 1.587E-04 | 7 |
| **34** | REACTOME_SCF_SKP2_MEDIATED_DEGRADATION_OF_P27_P21 | 5 | 2.282E-04 | 1 |
| **35** | KEGG_THYROID_CANCER | 4 | 2.461E-04 | 4 |
| **36** | REACTOME_G1_S_TRANSITION | 6 | 7.820E-04 | 1 |
| **37** | BIOCARTA_MCM_PATHWAY | 3 | 8.875E-04 | 1 |
| **38** | REACTOME_DOUBLE_STRAND_BREAK_REPAIR | 3 | 1.413E-03 | 8 |
| **39** | KEGG_NOTCH_SIGNALING_PATHWAY | 4 | 1.595E-03 | 7 |
| **40** | BIOCARTA_HER2_PATHWAY | 3 | 1.624E-03 | 6 |
| **41** | BIOCARTA_P53HYPOXIA_PATHWAY | 3 | 1.853E-03 | 5 |
| **42** | KEGG_NON_SMALL_CELL_LUNG_CANCER | 4 | 2.671E-03 | 4 |
| **43** | KEGG_GLIOMA | 4 | 5.230E-03 | 4 |
| **44** | KEGG_WNT_SIGNALING_PATHWAY | 6 | 5.700E-03 | 7 |
| **45** | REACTOME_GENERIC_TRANSCRIPTION_PATHWAY | 3 | 6.239E-03 | 2 |
| **46** | KEGG_MELANOMA | 4 | 7.149E-03 | 4 |
| **47** | KEGG_ADHERENS_JUNCTION | 4 | 1.036E-02 | 4 |
| **48** | KEGG_BLADDER_CANCER | 3 | 1.037E-02 | 4 |
| **49** | WNT_SIGNALING | 4 | 1.553E-02 | 7 |
| **50** | REACTOME_GENE_EXPRESSION | 10 | 1.569E-02 | 2 |
| **51** | REACTOME_ORC1_REMOVAL_FROM_CHROMATIN | 3 | 3.053E-02 | 1 |
| **52** | REACTOME_CELL_CYCLE_CHECKPOINTS | 4 | 3.102E-02 | 1 |
| **53** | KEGG_PPAR_SIGNALING_PATHWAY | 3 | 3.849E-02 | 3 |
| **54** | KEGG_PANCREATIC_CANCER | 3 | 3.990E-02 | 4 |
| **55** | KEGG_RENAL_CELL_CARCINOMA | 3 | 3.990E-02 | 4 |

*: 1: Cell Cycle; 2: Gene Expression; 3: Lipid Metabolism; 4: Cancer; 5: Apoptosis; 6: Signal transduction; 7: Development; 8: DNA Repair.
